# Combined magnetic fields provide robust coverage for interbody and posterolateral lumbar spinal fusion sites

**DOI:** 10.1007/s11517-015-1319-2

**Published:** 2015-06-05

**Authors:** Timothy Wade Stippick, Michael Richard Sheller

**Affiliations:** Escape Velocity Mechanical Design, 7229 S. Golfside Lane, Phoenix, AZ 85042 USA; Sheller Biomedical Innovations, 2517 N 61st Way, Scottsdale, AZ 85257 USA

**Keywords:** Lumbar vertebrae, Spinal fusion, Electromagnetic field (EMF), Electric field (EF), Magnetic flux density (MFD)

## Abstract

Electromagnetic fields generated by spinal bone growth stimulation devices have been computationally modelled to determine coverage of the lumbar spinal vertebrae. The underlying assumption of these models was that the electric field, but not the magnetic field, was therapeutically relevant. However, there are no published studies examining the therapeutic coverage of spinal fusion sites by stimulators utilizing combined magnetic fields. To assess the coverage, an anatomical model of the vertebrae and discs of the lumbar spine was developed to represent interbody and posterolateral fusion sites. Computer simulations of the induced electromagnetic fields were analysed to determine coverage of the fusion sites. For both interbody and posterolateral fusion models, combined magnetic fields provided 100 % coverage of the fusion sites for all intervertebral disc spaces and for all posterior planes from L1 to L5, respectively. Within the vertebral column, the magnitude of the electric field reached a maximum value of 3.6 × 10^−4^ V/m, which is several orders of magnitude less than any reported study demonstrating a biological effect. Given its clinical efficacy, a bone growth stimulator utilizing combined magnetic fields must rely on the action of its *magnetic* field rather than its *electric* field for a therapeutic effect.

## Introduction

Lumbar spine fusion procedures are commonly used to alleviate pain and suffering due to degenerative disc disease, spinal stenosis, displacement of disc without myelopathy, and acquired spondylolisthesis [41]. One of the parameters for a successful procedure is the fusion of the bone graft volume. The fusion rate is a multi-factorial process depending on instrumentation (none, rigid, semi-rigid), number of levels fused (1, 2, 3 or more), graft location (posterolateral, posterior interbody, anterior interbody), and graft source (autograft, allograft, synthetic) [5, 42]. Typical rates vary considerably depending on these factors and the surgical approach from 46 % success rate for a transforaminal interbody fusion [23] to 100 % for posterolateral lumbar interbody and posterolateral fusions [26, 32, 54]. Obviously, the fusion rates for patients with additional risk factors are significantly lower [27].

Given the number of patients undergoing lumbar spinal fusion procedures and the percentage of failed fusions, consideration of adjunctive therapies to further enhance the probability of fusion is warranted. Biophysical therapies utilizing stimulation by electromagnetic fields (EMFs) include combined magnetic field (CMF) and electric field (EF) devices. Furthermore, the generation of EMFs can be classified as direct current, capacitively coupled, and inductively coupled [48]. For patients using EMF devices, fusion rates increase an average of 18–32 % over controls [21, 28, 34, 37]. For example, in a study using a CMF device, Linnovitz et al. [34] reported a statistically significant 21 % increase in fusion rates compared with placebo. Criteria for inclusion were for primary, noninstrumented, intertransverse fusion of one or two vertebral levels.

Furthermore, the successful repair and consolidation of the fusion by an EMF device is directly correlated with the coverage of the spinal fusion volume by the stimulatory fields. Carter et al. [6] examined the current distribution of a capacitively coupled electric field device operating at 60 kHz and determined the magnitude of the input current to induce a biological response in a vertebral body. The anatomical model encompassed vertebrae from T5 to L5 and was derived from five computerized tomographic scans of the female abdomen. Another computational study investigated the current density generated by a capacitively coupled electric field device within a fracture of the spine, but did not draw any conclusions regarding the coverage of the spine by the EF [3]. Zborowski et al. [55] modelled the magnetic flux density (MFD) generated by a pulsed EMF device. Maxwell equations were solved using a piecewise analytical solution of the magnetic vector potential with an emphasis on visualization of the fields. The time derivative of the MFD was superimposed a posteriori onto a human spine. The underlying assumption of these models was that the EF, but not the MFD, was therapeutically relevant.

The CMF device in Linnovitz et al. [34] utilized an extremely low-frequency magnetic field (ELF-MF) combined with a static magnetic field to achieve clinical efficacy. The amplitude of the magnetic fields for the device was on the order of the earth’s magnetic field. The frequency was chosen to satisfy the ion cyclotron resonance theory for Ca^++^ and Mg^++^ [33]. Given this low amplitude and extremely low frequency, it is difficult to understand how the CMF signal could be perceived by a cell because of the thermally noisy environment surrounding it [1]. In fact, the interaction of Ca^++^ with an ELF-MF as prescribed by ion cyclotron resonance theory occurs at a much larger length scale (radius of gyration of the ion) than can be reconciled with distances associated with biochemical reactions [47].

A number of other biophysical theories have been advanced to elucidate the transduction of an externally applied ELF-MF into a local signal that can be detected at the level of an individual cell. Larmor precession [38, 39], radical pairs [49], F-actin-based Ca^2+^ signalling [20], electron tunnelling within enzymes [4], and Faraday coupling are the prevailing theories. In addition, the interaction of ions with ELF-MF contained within the coherence domains of water has been forwarded as a potential transduction mechanism [9, 13]. There is currently no consensus as to which theory best explains the coupling of ELF-MF to cellular phenomenon [17].

The transduction of an ELF-MF into a biochemical signal was demonstrated in early studies [14–16, 45]. For example, CMFs were shown to modulate the time course of insulin-like growth factor II (IGF-II) assayed at both 24 and 72 h post exposure in fracture callus obtained from the femurs of Sprague–Dawley rats [45]. More recent studies have described positive clinical effects on a diverse range of medical conditions and disease states [2, 8, 10, 12, 35, 44, 52]. However, these results are based on pilot studies and require additional clinical trials for substantiation. An ELF-MF limited osteoporosis due to spinal cord injury as indicated by improvements in bone mineral density and content as well as biochemical markers for bone including collagen I, osteocalcin, and alkaline phosphatase [36]. Finally, Ledda et al. [31] exposed human mesenchymal stem cells to a high-amplitude ELF-MF resulting in changes in cell morphology and increases in osteoblastic markers. The biological effects are likely due to both electric and magnetic field effects due to the high rate of change in the magnetic field with respect to time.

The purpose of this study was to model the therapeutic field associated with the only CMF device used as an adjunctive therapy for spinal fusion, the SpinaLogic^®^ bone growth stimulator. The model was first exercised to test the hypothesis that CMF provide targeted and complete coverage of lumbar spinal fusion sites for both interbody and posterolateral procedures. An additional hypothesis was tested to determine the validity of the assumption that the therapeutic effect of EMF stimulation for spinal fusions can only be attributed to the electric field.

## Methods

### Device description

**Fig. 1:**
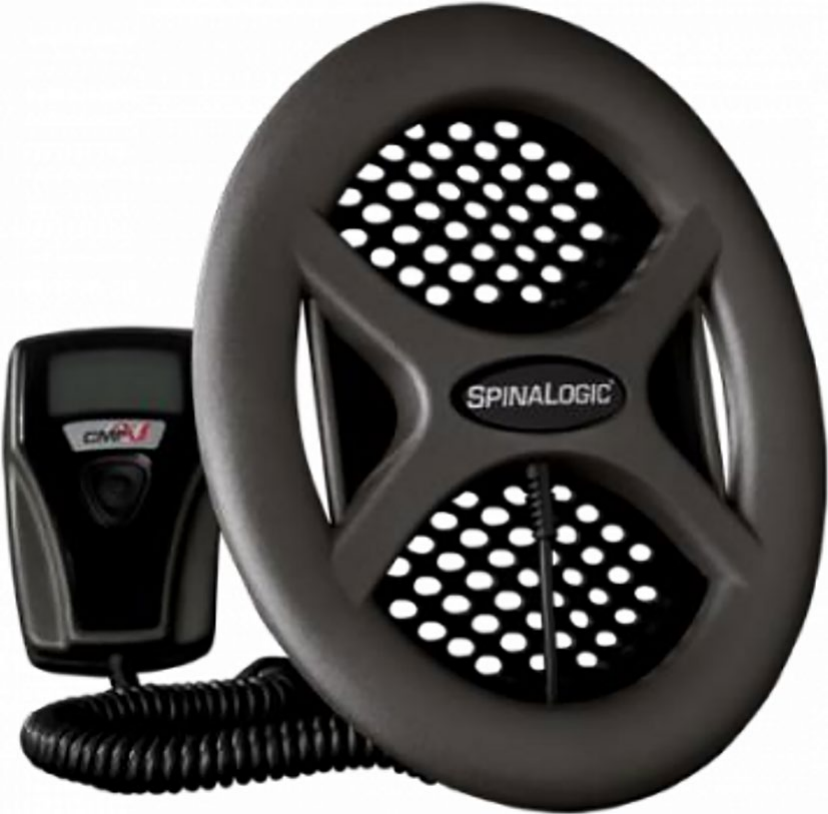
SpinaLogic^®^ bone growth stimulator. The electronic control module and the transducer coil are depicted. The module includes a signal generator that produces an electrical signal which is transmitted to the treatment transducer. The transducer coil is an elliptically shaped copper wire coil that converts the electrical signal into an electromagnetic field

**Fig. 2:**
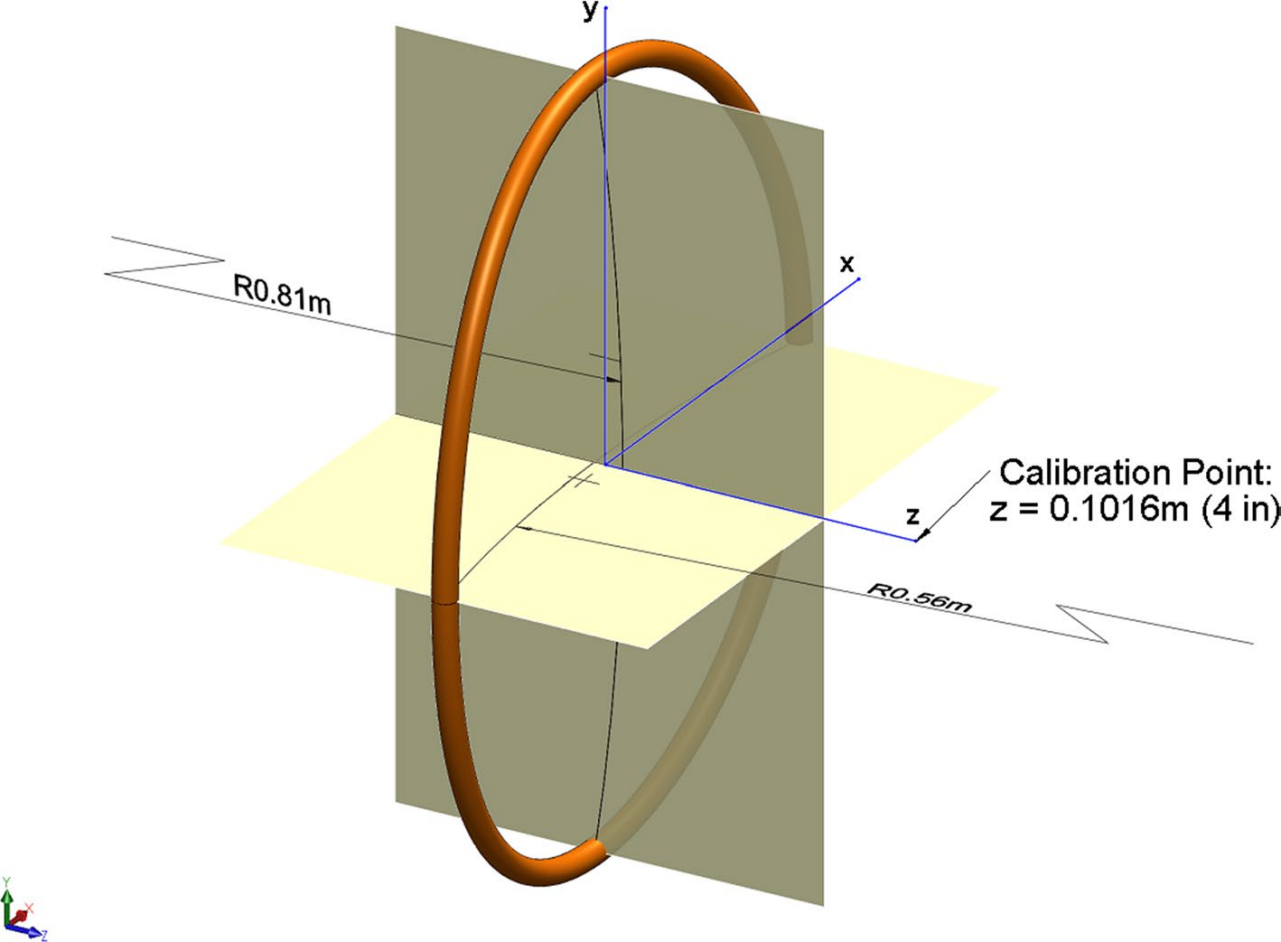
Model of SpinaLogic^®^ transducer coil. The *x* and *y* axes were chosen to coincide with the minor and major axes of the coil, respectively

The SpinaLogic^®^ bone growth stimulator (DJO, Vista, CA) consists of a three-dimensional patient interface contoured to follow the curvature of the lumbar spine, a control box containing the electronics, and a battery pack (Fig. 1). The device is used for 30 consecutive minutes a day until fusion occurs as determined by the treating physician. The patient interface consists of a single transducer coil to generate the magnetic field and a magnetoresistive element to provide feedback as to the magnitude of the earth’s magnetic field which ranges from 25 to 65 microTesla (μT). The coil is constructed using 504 turns of 30-gauge copper magnetic wire. Its projection in two dimensions is an ellipse with a major axis of 0.24 m and a minor axis of 0.19 m. When viewed from the side, the coil forms a circular arc with an approximate radius of 0.81 m, and, when viewed from the top, the coil forms a circular arc with an approximate radius of 0.56 m (Fig. 2).

When the specified current is applied to the coil, it generates an extremely low-frequency and extremely low-intensity magnetic field that has both alternating and direct current (AC and DC) components, labelled a combined magnetic field (CMF). Specifically, the field oscillates sinusoidally at a frequency of 76.6 Hertz with an AC component of 40.0 ± 8.0 μT, peak to peak, and a DC component of 20.0 ± 2.0 μT and is calibrated at a distance of 0.10 m normal to the projected plane of the coil (see Fig. 2). The tolerances for the AC and DC fields are applied to the component of the MFD, $B_{z}$, normal to the plane of the coil. It is important to note that specifications based on these tolerances (AC: 32.0–48.0 μT, DC: 18.0–22.0 μT) are not the same as the limits of the therapeutic field, which are currently unknown.

Clinical data from Linnovitz et al. [34] were used to estimate the lower limit for the magnitude of the MFD, $\left| {\vec{B}} \right|$, that was efficacious. First, the soft tissue distance to the intervertebral disc space for L1–L2 was calculated based on regression equations derived from renal scintigraphy (the kidneys are located adjacent to the disc space) [46]. Since most of the spinal fusions in the study were located at the L4–L5 disc level, an adjustment was made to account for the additional depth from L1–L2 to the L4–L5 disc space based on the anatomical CAD model. The depth was also augmented to account for the cross section of the coil and packaging for the device. Finally, the MFD for the trapezoidal coil tested in the clinical trial was modelled using the Biot–Savart law [43]. The magnitude of the MFD to L4–L5 was then calculated at the L4–L5 depth resulting in a lower limit for $\left| {\vec{B}} \right|$ of 22.0 μT. No upper limit was assumed for the MFD. Thus, the specification for a therapeutic magnetic field was any value of the MFD above the lower limit.

### Anatomical spine model

An anatomically based CAD model of the lumbar spine of a 50th percentile female was adapted for use in the simulations (BodyWorks, NZ, http://www.zetec.co.nz/bodyworks/). A female lumbar spine model was used because of its availability and the extensive rework that would be involved in rescaling it to represent the male lumbar spine. Other studies have also used the female spine in their modelling [6]. The model contains the L1 through L5 vertebrae and their associated intervertebral discs. Dimensions for the lumbar vertebrae were obtained from the literature [11, 53, 56, 57]. The interbody fusion model was created by replacing the tissue within the intervertebral spaces with blood as the graft material. The posterolateral fusion model was created by reducing the bone on the posterior faces to simulate decortication and replacing the tissue with blood [50]. The dielectric properties of cortical bone, cancellous bone, and soft tissues were specified in terms of conductivity and permittivity with values obtained from the literature [18, 19, 51].

### Electromagnetic field simulation

ElectroMagnetic Simulation (EMS) software was used for simulating the magnetic and electric fields (ElectroMagneticWorks, Montreal, Quebec, Canada). The software was chosen because it is fully embedded in SolidWorks (Dassault Systèmes SolidWorks Corp., Waltham, Massachusetts), the same software that was used to create the SpinaLogic^®^ and anatomical spine CAD models. EMS uses the finite element method which is also compatible with the complexity of the spine model. The transient magnetic analysis option was chosen for magnetic field simulations in order to model simultaneously both the AC and DC components of the magnetic field. The computations for the transient analysis were run until steady state was reached.

**Fig. 3:**
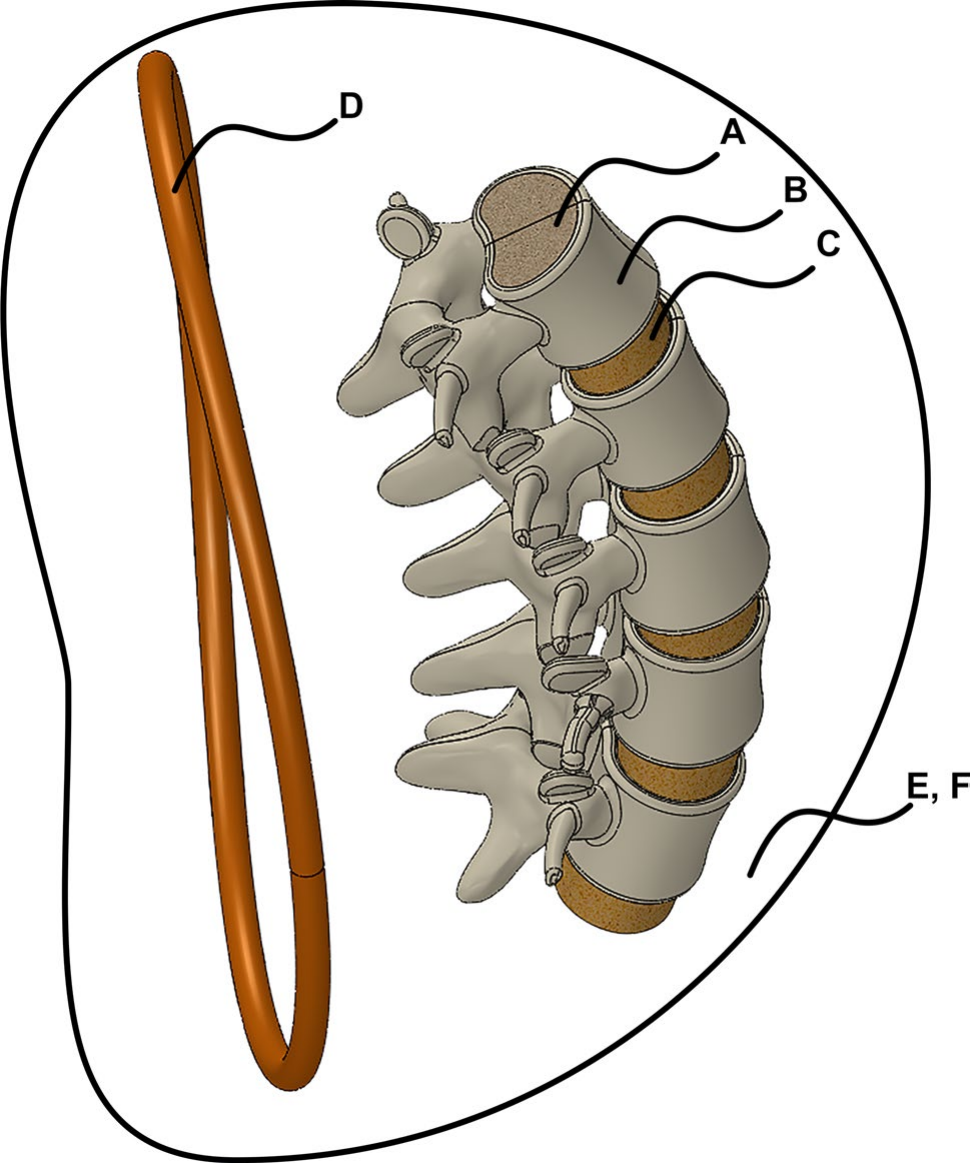
Tissues modelled in anatomical model and surrounding medium

For low-frequency electromagnetic fields, displacement currents are neglected (magneto-quasistatic analysis), and the electric field is calculated directly from the time harmonic form of the Maxwell–Faraday equation given by $\nabla \times \vec{E} = - j\omega \vec{B}$. The AC magnetic analysis option was used because the EF depends only on the AC magnetic field. The relative magnetic permeability, electrical permittivity, and electrical conductivities for cortical bone, cancellous bone, blood, and the surrounding medium (air and all other soft tissues) were specified for the transient and AC magnetic analyses as listed in Tables 1 and 2 for the tissues shown in Fig. 3. The electrical conductivity is required to compute the current density within the coil, whereas only the magnetic and electric fields were computed for the spine model. To verify the EMS simulations, all fields were also calculated using a magnetic vector potential approach for an equivalent elliptical coil [7, 24] and programmed in MathCad (PTC, Needham, MA).

**Table 1:** Material properties for transient analysis

Fig. ID	Component	Material type	Permittivity	Relative permeability	Electrical conductivity (S/m)
A	Vertebrae, inner	Cancellous bone	8.0E5	1	0.1
B	Vertebrae, outer	Cortical bone	3.162E3	1	0.016
C	Disc	Blood	3.162E3	1	0.66
D	Coil	Copper	1.0	0.999991	5.7E7
E	Surrounding medium, inner/near	Air	1.0	1	0.0
F	Surrounding medium, outer/far	Air	1.0	1	0.0

**Table 2:** Material properties for AC analysis

Fig. ID	Component	Material type	Permittivity	Relative permeability	Electrical conductivity (S/m)
A	Vertebrae, inner	Cancellous bone	8.0E5	1	20
B	Vertebrae, outer	Cortical bone	3.162E3	1	20
C	Disc	Blood	8.0E5	1	20
D	Coil	Copper	1.0	0.999991	5.7E7
E	Surrounding medium, inner/near	N/A	8.0E5	1	20
F	Surrounding medium, outer/far	N/A	8.0E5	1	20

To provide an estimate of the far-field boundary conditions, the tangential and normal components of the MFD, $\vec{B}\left( {x,y,z} \right)$, for the elliptical coil were calculated to determine the distances where their magnitudes were less than 0.5 μT. Since the curvature of the SpinaLogic^®^ coil along the major and minor axes is relatively small, this approximation was deemed adequate for the simulations. Moreover, a sensitivity analysis was conducted by varying the location of the far-field boundary conditions, checking the difference introduced at the calibration point, and finally comparing the solution along the *z*-axis to the magnetic vector potential solution for the elliptical coil.

To summarize, a realistic anatomical model was developed for both interbody and posterolateral fusion procedures. The CMF generated by the SpinaLogic^®^ transducer coil was then computationally modelled to assess whether the field exceeded the putative therapeutic limit within the fusion site. Finally, the EF was computed and compared with the open literature to determine whether its amplitude exceeded the threshold for a biological response.

## Results

**Fig. 4:**
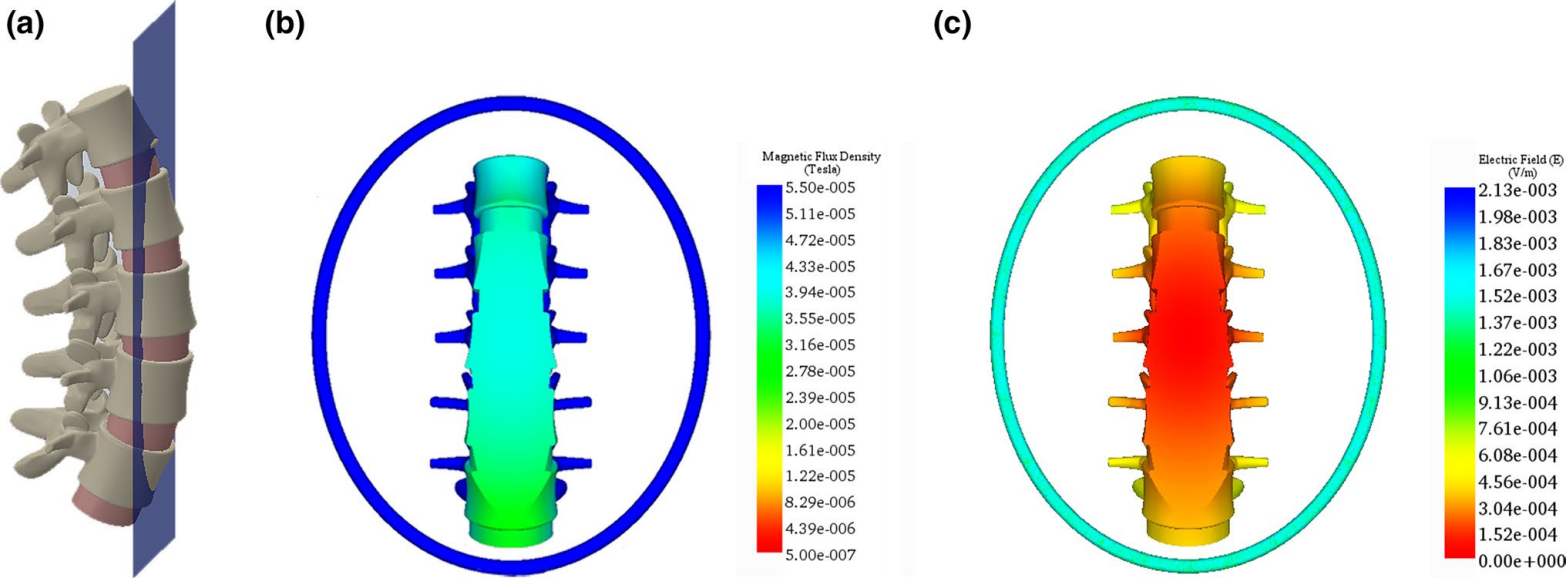
Simulation of the magnetic flux density and electric field for the coronal plane at *z* = 0.1016 m (calibration plane): **a** anatomical model, **b** $\left| {\vec{\varvec{B}}} \right|$, magnitude of the magnetic flux density, and **c** $\left| {\vec{\varvec{E}}} \right|$, magnitude of the induced electric field. $\left| {\vec{\varvec{B}}} \right|$ exceeds the lower limit of 22.0 μT for the entire plane, and $\left| {\vec{\varvec{E}}} \right|$ is lower than the threshold for a biological effect. In **b** and **c**, the coil is 0.10 m in front of the spine

**Fig. 5:**
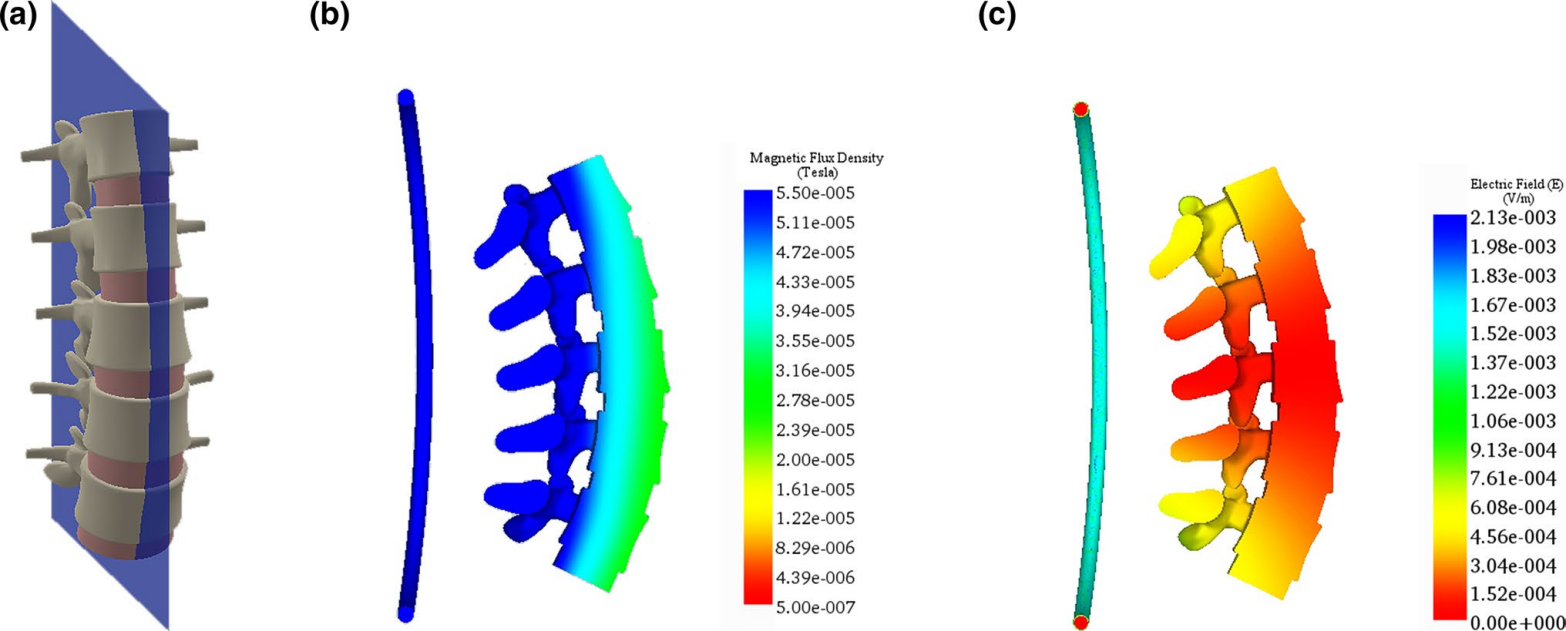
Simulation of the magnetic flux density and electric field for the mid-sagittal plane: **a** anatomical model, **b** $\left| {\vec{B}} \right|$, magnitude of the magnetic flux density, and **c** $\left| {\vec{E}} \right|$, magnitude of the induced electric field. $\left| {\vec{B}} \right|$ exceeds the lower limit of 22.0 μT for the entire plane, and $\left| {\vec{E}} \right|$ is lower than the threshold for a biological effect

The SpinaLogic^®^ coil and a realistic anatomical model of the lumbar spine were efficiently meshed (Figs. 4a, 5a), and simulations of the EMF were generated for both interbody and posterolateral fusion procedures. The MFD was unchanged by the introduction of the anatomical model because the magnetic permeability of these tissues is essentially the same as free space. The simulation of $\left| {\vec{B}} \right|$ for the coronal plane at *z* = 0.1016 m (the calibration plane) and the mid-sagittal plane is shown in Figs. 4b and 5b, respectively. The depth of penetration corresponding to the lower limit of 22.0 μT is approximately 0.1524 m. The simulation of $\left| {\vec{E}} \right|$ for the calibration plane and the mid-sagittal plane is shown in Figs. 4c and 5c, respectively. The maximum amplitude of the electric field was 3.6*10^−4^ V/m in the graft tissue within the calibration plane. There are no known biological effects at these amplitudes [25].

### Magnetic field simulation for interbody fusion

**Fig. 6:**
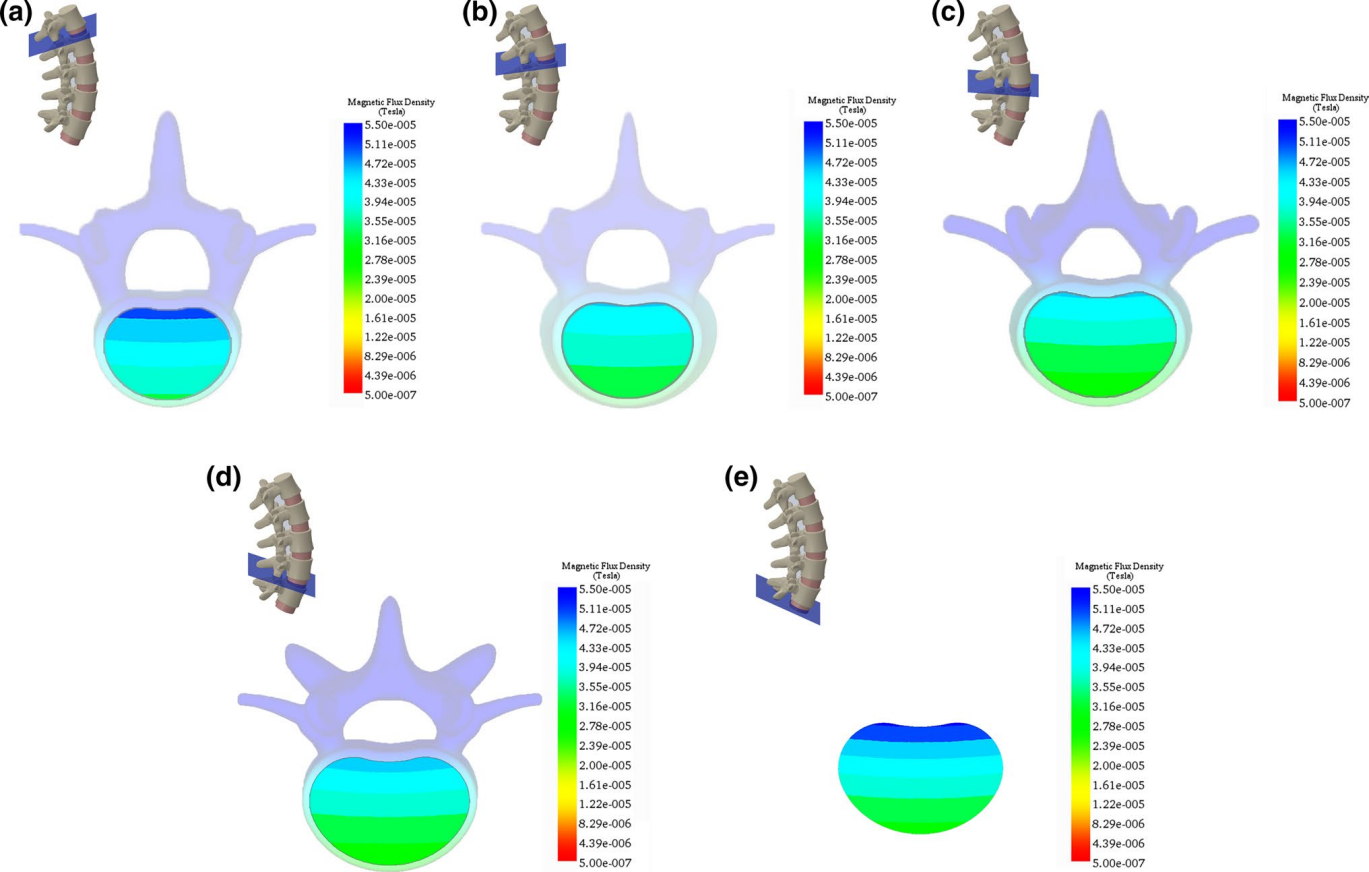
Simulation of the magnitude of the magnetic flux density, $\left| {\vec{\varvec{B}}} \right|$, for the transverse planes bisecting the intervertebral disc space between: **a** L1–L2, **b** L2–L3, **c** L3–L4, **d** L4–L5, and **e** L5–S1. $\left| {\vec{\varvec{B}}} \right|$ exceeds the lower limit of 22.0 μT for each plane providing 100 % coverage of the interbody fusion site. The inferior vertebra is shown in **a** through **d**

For the interbody fusion, simulation of $\left| {\vec{B}} \right|$ for the transverse planes intersecting the vertebral column is shown in the insets in Fig. 6. For example, for the L1–L2 transverse plane, the plane was positioned midway between the inferior endplate of the L1 vertebra and superior endplate of the L2 vertebra at the appropriate lordotic angle [11]. The plane was chosen to bisect the intervertebral disc space where the graft material is placed. The in-specification $\left| {\vec{B}} \right|$ for this plane provided areal coverage of 100 % (Fig. 6a). Similarly, the in-specification $\left| {\vec{B}} \right|$ for the transverse planes bisecting L2–L3, L3–L4, L4–L5, and L5–S1 provided areal coverages of 100 % for all planes (Fig. 6b–e).

### Magnetic field simulation for posterolateral fusion

**Fig. 7:**
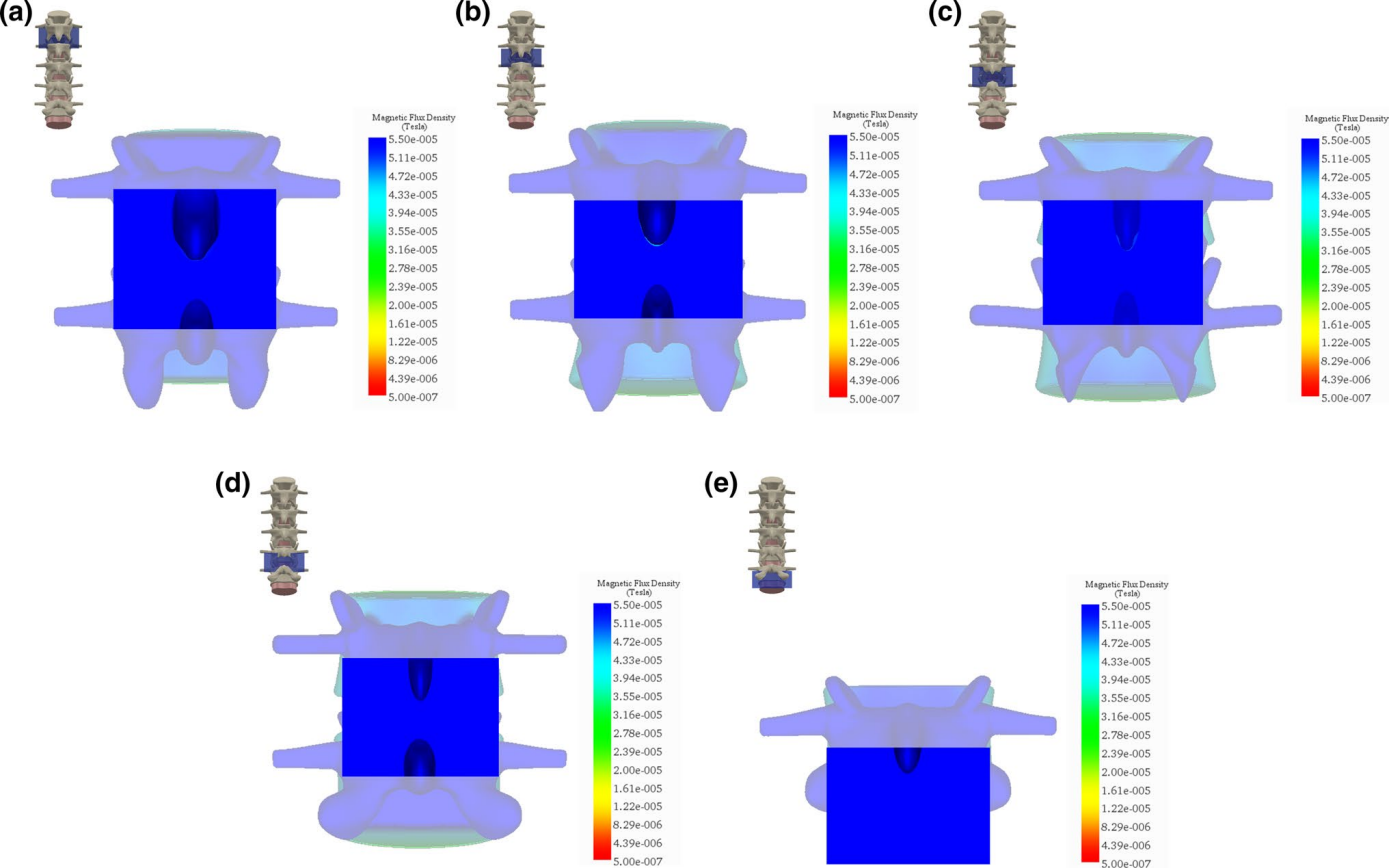
Simulation of the magnetic flux density, $\left| {\vec{\varvec{B}}} \right|$, for the posterolateral planes spanning the intervertebral disc space between: **a** L1–L2, **b** L2–L3, **c** L3–L4, **d** L4–L5, and **e** L5–S1. $\left| {\vec{\varvec{B}}} \right|$ exceeds the lower limit of 22.0 μT for each plane providing 100 % coverage of the posterolateral fusion site

For the posterolateral fusion, simulation of $\left| {\vec{B}} \right|$ for the posterior planes paralleling the vertebral column is shown in the insets in Fig. 7. These planes were chosen to span the fusion area for each intervertebral space from L1 to L5. For example, a posterior plane was positioned to span the intervertebral space from L1 to L2 where the graft material is placed. The in-specification $\left| {\vec{B}} \right|$ for this plane surpassed the lower limit with minimum and maximum values of 67.5 and 78.0 μT (Fig. 7a). Similarly, the planes spanning L2–L3, L3–L4, L4–L5, and L5–S1 surpassed the lower limit with minimum values of 60.0, 60.5, 61.5, and 68.5 μT, respectively, and maximum values of 67.0, 65.0, 69.5, and 93.0 μT, respectively (Fig. 7b–e).

## Discussion

Realistic anatomical models are necessary when determining the coverage of the bone graft material for lumbar spine fusion sites by a medical device using EMF. For this purpose, an anatomical model of the lumbar spine was developed, and the EMF generated by the SpinaLogic^®^ was simulated to determine the coverage of the graft material for both interbody and posterolateral spinal fusion procedures involving the L1–L5 vertebrae. The simulations indicate that there is 100 % coverage by the therapeutic magnetic field of all spinal fusion sites. To the authors’ knowledge, there are no other published studies of extremely low-frequency and extremely low-intensity magnetic field applied to spinal fusion procedures.

Critical to the accuracy of the simulations was the selection of the boundary conditions. The MFD for the far-field boundary condition was determined first by numerical integration of the Biot–Savart law as applied to an elliptical coil in air. For the SpinaLogic^®^ simulations, the normal and tangential components were set to zero at the distance calculated for the elliptical coil, which introduced absolute errors of approximately 0.5 μT at the boundaries. At the calibration point, this translated to an error of 1.25 % or less. This first exercise of the model in air validated its use for the more complex simulations of single and multilevel fusions.

The introduction of the anatomical model of the spine for the L1–L5 vertebrae did not affect the magnitude of the MFD. Thus, every point within the fusion volume realized the effects of the magnetic field, which is a fundamental property of magnetic fields when interacting with nonmagnetic material. From a biological standpoint, this property allows CMF to stimulate all cells and their intracellular components within the treatment volume required for bone repair or consolidation. Furthermore, since the magnitude includes all three components of the magnetic field, the in-specification area associated with $\left| {\vec{B}} \right|$ exceeds specifications based on these tolerances for $B_{z}$. Thus, greater areal coverage is provided by $\left| {\vec{B}} \right|$ than is currently attributed to the SpinaLogic^®^ device. Based on biophysical principles, it can be argued that bone cells involved with the repair and consolidation of the fusion volume are stimulated by the magnitude, not by a single component, of the combined magnetic fields.

The limits for the MFD were critical in determining the coverage of the fusion sites. For the lower therapeutic limit, clinical data were used to estimate the thickness of the soft tissues to the L4–L5 space, which is where most of the fusions occurred in the CMF spinal fusion study. The assumption is that the MFD at this depth of penetration accounted for the successful fusions in the treated population. However, the lower limit for a therapeutic effect is currently unknown, so this derived limit is speculative at best. In lieu of additional clinical research, this approach was considered to be better than basing the therapeutic field on engineering tolerances. Biological effects have been noted for time-varying magnetic fields at 1.0 μT [49]. For the upper limit, a higher MFD is presumed to be therapeutically beneficial because an increase in intensity of this magnitude most likely leads to a higher probability of receptor–ligand binding [38]. Further clinical studies are needed to determine the full range of magnetic field intensities that are therapeutically beneficial.

For the interbody fusion, the areal sections from the simulation demonstrated that the entire fusion volume was stimulated by the in-specification magnetic field. The area in-specification covered 100 % of the area involved in the fusion for the transverse planes associated with the intervertebral spaces. Thus, the depth of penetration of the magnetic field was sufficient to stimulate the entire bone graft volume from the anterior to the posterior surfaces for all lumbar vertebrae. Likewise, the areal sections from the simulation of the posterolateral fusion demonstrated that the entire L1–L5 fusion volume was 100 % covered by the magnetic field. The MFD ranged from 60.0 to 93.0 μT for all posterior planes. Thus, the height and width of the magnetic field were sufficient to stimulate the entire bone graft volume for all lumbar vertebrae. The simulation of these two very different fusion procedures supports the assertion that the coverage by the magnetic field of the SpinaLogic^®^ is extremely robust.

The magnitudes of the electric field for the simulations of both the anterior interbody and posterolateral fusion were below thresholds known to elicit a biological effect [25]. This is not surprising because the SpinaLogic^®^ bone growth stimulator was designed to provide combined magnetic fields at an extremely low frequency of 76.6 Hz and at an extremely low intensity of 40.0 μT, peak to peak, with a 20.0-μT DC offset. Therefore, the electric field does not contribute to the therapeutic effect, and the efficacy of the SpinaLogic^®^ must depend on the interaction of cells with the CMF (or on some yet unknown exotic physical phenomenon). This is somewhat surprising since most EMF-based bone growth stimulators are thought to depend on electrical stimulation to achieve their effect [40]. However, it should be noted that there are currently multiple biophysical mechanisms that have been proposed that couple low-intensity ELF-MF to molecular/cellular phenomenon [9, 17].

Although the biophysical mechanism is unknown, the site of transduction of the magnetic field by an individual cell may be conjectured. Gartzke and Lange [20] argue for direct energy transfer from ELF-MF to Ca^++^ at the associated ion cyclotron resonance frequency in microvilli located on the cell surface. Filamentous actin mediates Ca^++^ signalling within the microvilli by limiting diffusion of divalent cations and providing a store for Ca^++^ [30]. Cations are loosely bound to the anionic charge centres along F-actin bundles and can flow when exposed to low-intensity ELF-MF because each cation is acted upon simultaneously by the magnetic field. Further experiments using CMF to explore Ca^++^ signalling along F-actin bundles are warranted especially ones that manipulate the states of the microvilli. However, it should be noted that F-actin itself may be effected by ELF-MF. Human mesenchymal cells exposed to a nonpulsed, sinusoidal EMF using a high-amplitude alternating field (1 mT) at 50 Hz induced drastic changes in cell morphology as evidenced by increased actin formation and redistribution [31].

The time course of insulin-like growth factors and their related binding proteins (IGFs and IGFBPs) was recently investigated in a nonunion model in the rat [29]. The model was created by cauterizing the periosteum after fracture and is expected to successfully reproduce a nonunion. IGF-II was significantly upregulated in nonunions compared with normal healing fractures at 7 days postfracture. In addition, IGFBP-6 was upregulated at 7, 14, and 28 days postfracture and also plays a major role in inhibiting osteoblast differentiation [22]. Ryaby et al. [45] assumed that stimulation of IGF-II by CMF promoted the healing of nonunions and spinal fusions in clinical trials. However, IGF-II was also downregulated at day 14 postfracture in the same study. Thus, the therapeutic effect of CMF may be to modulate IGF-II directly or to downregulate IGFB-6 at the appropriate time points. Using the same nonunion and normal fracture healing models, experiments could be conducted to elucidate the role of CMF in modulating IGF-II and its binding proteins.

## Conclusions

Based on the modelling completed for this study, the CMF generated by the SpinaLogic^®^ provides targeted and complete coverage for both interbody and posterolateral spinal fusion procedures involving the L1–L5 vertebrae. The SpinaLogic^®^ bone growth stimulator utilizes CMF that can be characterized as extremely low-frequency and extremely low-intensity magnetic fields. The amplitude of the associated electric field is less than the thresholds for a biological effect [25]. However, clinical experience supports the efficacy of the SpinaLogic^®^ stimulator when compared with other bone growth stimulators that employ electromagnetic fields. Therefore, CMF must interact with bone graft material via magnetic fields to promote repair and consolidation of the fusion volume. Moreover, the coverage by the SpinaLogic^®^ is more than adequate to stimulate multi-level spinal fusions. Indeed, simulations of the magnetic field for both interbody and posterolateral fusions show that 100 % of the fusion volumes are stimulated.
